# Histological response of gastric adenocarcinomas after chemotherapy in the Tunisian population

**DOI:** 10.1590/0102-67202025000032e1901

**Published:** 2025-10-03

**Authors:** Dhouha BACHA, Ines MALLEK, Sarra BEN-REJEB, Monia ATTIA, Lassaad GHARBI, Ahlem LAHMAR, Sana BEN-SLAMA

**Affiliations:** 1 Mongi Slim University Hospital, Department of Pathology – Tunis, Tunisia.; 2 Laboratory Head at Security Forces Hospital, Department of Pathology – Tunis, Tunisia.; 3 Mongi Slim University Hospital, Department of Radiology – Tunis, Tunisia.; 4 Mongi Slim University Hospital, Department of Surgery – Tunis, Tunisia.

**Keywords:** Adenocarcinoma, Stomach, Response Elements, Drug Therapy, Histology, Prognosis, Adenocarcinoma, Estômago, Elementos de Resposta, Tratamento Farmacológico, Histologia, Prognóstico

## Abstract

**Background:**

Gastric cancer is the fifth most common and a leading cause of cancer death. Since 2005, perioperative chemotherapy (CT) has been the standard for non-metastatic gastric adenocarcinomas. Tumor response relies essentially on histological criteria.

**Aims:**

The aim of the study was to evaluate tumor regression grade (TRG) after neoadjuvant CT and compare the Mandard and Becker scoring systems.

**Methods:**

This 15-year retrospective study included patients with gastric adenocarcinoma treated with neoadjuvant CT and surgery. The TRG was assessed using Mandard and Becker scores, evaluated by area under the curve (AUC) for homogeneity, monotonicity, and discrimination. Tumors were staged by the American Joint Committee on Cancer and classified as the World Health Organization.

**Results:**

Forty patients (mean age 62 years; M:F ratio 2.6) were included. Tubular adenocarcinoma was the most common (48%), and 20% were stage IV. Mandard TRG1 and TRG5 each accounted for 15%, with median survivals of 48 and 30.5 months, respectively. For Becker TRG, they were 25.15 months (TRG 1), 24 months (TRG 2), and 54 months (TRG 3). The mean survival was 49.2 months for TRG1 and 39.2 months for TRG5 (Mandard), 50.3 months for TRG1 and 42.2 months for TRG3 (Becker). The positive predictive values for Mandard and Becker were 1.116 and 0.418 at 1 year and 5.719 and 1.820 at 5 years. The linearity values for Mandard and Becker were 0.6 and 0.3 at 1 year and 2.5 and 2.2 at 5 years. The AUC values at 1 year were 0.568 (Mandard), and 0.545 (Becker), and 0.606 for both at 5 years.

**Conclusions:**

TRG is an independent survival predictor in gastric cancer, with similar performance between Mandard and Becker scores. Combined with ypTNM staging, it may enhance prognostic accuracy.

## ARTICLE HIGHLIGHTS

This 15-year retrospective study compares the prognostic performance of the Mandard and Becker tumor regression grading systems in gastric adenocarcinoma.

Both tumor regression grade systems showed moderate and comparable predictive value for 1- and 5-year overall survival.

The Mandard score showed slightly better linearity and positive predictive value, but no clear superiority was observed.

Combining tumor regression grade classification with ypTNM staging may improve prognostic accuracy in gastric cancer patients treated with neoadjuvant chemotherapy.

## Summary

**CENTRAL MESSAGE:**

Gastric cancer is the 5th most common cancer globally and the 4th leading cause of cancer-related deaths. Since 2005, perioperative chemotherapy has been the standard for non-metastatic gastric adenocarcinomas. Tumor response relies essentially on histological criteria, with many scoring systems, the most used of which are Mandard and Becker, but without consensus.

**PERSPECTIVES:**

This study confirms that the tumor regression classification is an independent prognostic factor in gastric cancer, particularly when combined with ypTNM staging. While Mandard shows slightly better performance, both Mandard and Becker scores demonstrate moderate and comparable predictive value, with no clear superiority. These findings highlight the need for a standardized system integrating nodal response and other key histological features to improve prognostic accuracy.

## VISUAL ABSTRACT

**Figure:**
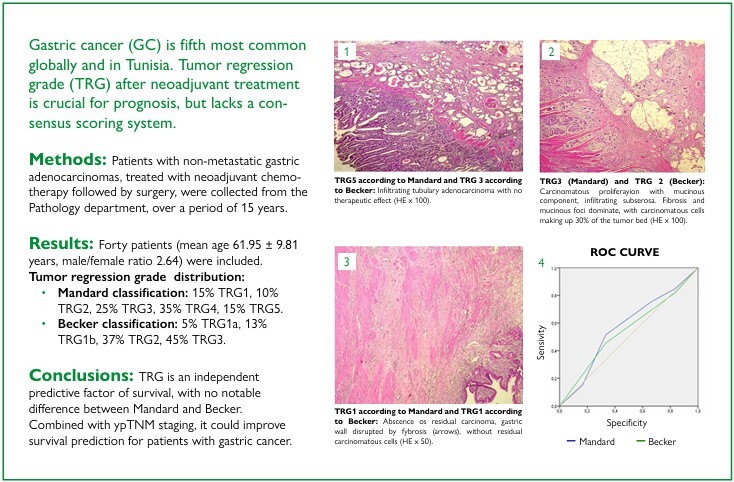

## RESUMO

**Racional:**

O câncer gástrico é o quinto mais comum e uma das principais causas de morte por câncer no mundo. Desde 2005, a quimioterapia perioperatória (QT) tem sido o padrão para adenocarcinomas gástricos não metastáticos. A resposta tumoral depende essencialmente de critérios histológicos.

**Objetivos:**

Avaliar a regressão tumoral após quimioterapia neoadjuvante e comparar os sistemas de pontuação de Mandard e Becker.

**Métodos:**

Estudo retrospectivo de 15 anos com pacientes com adenocarcinoma gástrico tratados com quimioterapia neoadjuvante seguida de cirurgia. A regressão tumoral (RGT) foi avaliada pelos escores de Mandard e Becker, com análise da homogeneidade, monotonicidade e capacidade discriminativa por meio da AUC. A classificação seguiu o Comitê Conjunto Americano sobre Câncer e classificado conforme a Organização Mundial de Saúde.

**Resultados:**

Foram incluídos 40 pacientes (idade média: 62 anos; razão H:M de 2,6). O adenocarcinoma tubular foi o tipo mais comum (48%) e 20% estavam em estágio IV. Pelo escore de Mandard, 15% foram RGT 1 e 15% RGT 5, com sobrevidas medianas de 48 e 30,5 meses. No escore de Becker, as sobrevidas medianas foram de 25,15 meses (RGT1), 24 meses (RGT2) e 54 meses (RGT3). As sobrevidas médias foram de 49,2 meses (RGT1) e 39,2 meses (RGT5) para Mandard; 50,3 meses (RGT1) e 42,2 meses (RGT3) para Becker. Os valores preditivos positivos aos 12 meses foram 1,116 (Mandard) e 0,418 (Becker); aos 5 anos, 5,719 e 1,820. AUC: 0,568 (Mandard) e 0,545 (Becker) em 1 ano; 0,606 para ambos em 5 anos.

**Conclusões:**

A regressão tumoral é um preditor independente de sobrevida no câncer gástrico. Os escores de Mandard e Becker têm desempenho semelhante e, combinados ao estadiamento ypTNM, podem melhorar a previsão prognóstica.

## INTRODUCTION

 Gastric cancer (GC) is the 5th most common cancer globally and the 4th leading cause of cancer-related deaths^21^. In Tunisia, GC ranked 8th among diagnosed cancers and was the 4th leading cause of cancer deaths, accounting for 7.7% of mortality-related cancer^8^. Despite advances, GC prognosis remains poor, with a 5-year survival rate under 30%^1^. Since 2005, perioperative chemotherapy (CT) has been the standard for non-metastatic gastric adenocarcinomas (GADC)^22^. Tumor response relies essentially on histological criteria, with many scoring systems, the most used of which are Mandard and Becker, but without consensus^19^. 

 This study aims to evaluate histological responses to neoadjuvant CT in GADC using the Mandard and Becker systems^3,12^. 

## METHODS

 This retrospective study included patients with non-metastatic GADC treated with neoadjuvant CT and surgery at Mongi Slim Hospital, La Marsa (North of Tunisia) from July 2008 to July 2023. Cases were collected from the Pathology Laboratory, and surgeries were performed in the Visceral Surgery Department. Exclusion criteria were non-adenocarcinoma types (e.g., lymphomas, neuroendocrine tumors), patients diagnosed via biopsy only, those who did not undergo surgery, and cases without prior neoadjuvant CT. Patients with esophagogastric junction adenocarcinoma or missing clinical records were also excluded. 

 All patients in the study received the FLOT protocol^14,15^, which consists of 5-fluorouracil, leucovorin, oxaliplatin, and docetaxel. On average, patients underwent 4.17±1.11 cycles of treatment, with the number of cycles ranging from 2 to 6. 

### Collection of data

 Clinical and pathological data were collected from patient records. Histological classification followed Laurén’s 1965 system^2^ and World Health Organization (WHO’s 5th edition)^13^, with staging per American Joint Committee on Cancer (AJCC pTNM, 8th edition)^6^. Tumor regression grade (TRG) was assessed using Mandard and Becker scoring systems^3,12^. Mandard TRG ranges from TRG 1 (no residual cancer, complete fibrosis) to TRG 5 (no regression). Becker TRG groups include TRG 1 (0–<10% residual tumor), TRG 2 (10–50%), and TRG 3 (>50% residual tumor). The study was approved by the Ethics Committee of the Institution (number 08/2024). 

### Statistical analysis

 Data were analyzed using SPSS® (Statistical Package for the Social Sciences) version 24.0. Qualitative variables were reported as frequencies, and quantitative variables as means with ranges. Mortality was assessed with Kaplan-Meier survival curves and compared using the log-rank test. Mandard and Becker scores were evaluated for homogeneity (likelihood ratio: LR), monotonicity (linear trend: LT), and discriminatory capacity with the area under the curve (AUC). Higher LR+, LT, and AUC values indicated better TRG score performance in predicting survival. Statistical significance was set at p<0.05. 

## RESULTS

 This study included 40 patients, with an average age of 61.95±9.81 years (range: 40–77, median: 63). The cohort comprised 29 men and 11 women, with a male-to-female ratio of 2.64. Diagnostic delay averaged 7 months. The main clinicopathological findings are summarized in Table 1. 

**Table 1:** The main clinicopathological findings.

Features		Number of cases (n)	Percentage (%)
Clinical symptoms			
	Epigastric pain	34	85
	Poor general condition (weight loss, fatigue)	34	85
	Anemia	13	32.5
Physical examination			
	Normal	22	55
	Abdominal tenderness	18	45
	Epigastric mass	2	5
Tumor sites			
	Antrum	13	32
	Subcardial	8	20
	Gastric body	4	10
	Cardia	4	19
	Lesser curvature	3	7.5
	Entire stomach	2	5
	Antro-pyloric	2	5
	Fundus	2	5
	Greater curvature	1	2.5
Bormann endoscopic features			
	Type I (mass)	2	5
	Type II (ulcerative)	6	15
	Type III (infiltrative ulcerative)	27	67
	Type IV (diffuse infiltrative)	5	13
Type of gastrectomy			
	Total	32	80
	Subtotal (4/5)	8	20
Lymph node resection			
	D1.5	30	75
	D2	10	25
Resection margins			
	R0	36	90
	R1	4	10
Histological subtypes			
	Intestinal	16	40
	Diffuse	16	40
	Mixed	8	20
Tumor differentiation			
	Well	13	32.5
	Moderate	10	25
	Poor	17	42.5
Post-operative stage			
	IA	5	12.5
	IB	4	10
	IIA	5	12.5
	IIB	7	17.5
	IIIA	6	15
	IIIB	3	7.5
	IIIC	2	5
	IV	8	20

D: lymph node dissection; R0: complete ressection

On pathological findings, the average tumor size was 42.34±17.44 mm (range: 15–140 mm).

 Lymphovascular and perineural invasion were frequently observed, with lymphatic emboli present in 42% of cases and perineural invasion identified in 32%. 

### Postoperative outcomes

 Postoperative evolution was uncomplicated in the majority of patients (90%). However, four patients (10%) required surgical revision due to complications such as hemoperitoneum (one case), fistula (one case), or intestinal obstruction (two cases). 

### Recurrence patterns

 Local recurrence was observed in 17% of patients, with a mean time to recurrence of 16.86 months. Metastatic recurrence occurred in 23% of cases, with a mean delay of 21.25 months following surgery. 

### Survival outcomes

 Disease-free survival (DFS) rates declined sharply over time, with 42% at 1 year, 20% at 3 years, and only 10% at 5 years. Among the 40 patients included, 38% died during the study period, with a mean age at death of 60.08 years and a mean survival of 22 months. 

### Overall survival and prognostic implications

 The overall survival (OS) rates were 84.6% at 1 year, 53.8% at 3 years, and 31.8% at 5 years. These figures underscore the aggressive nature of GADC and its significant impact on long-term survival, despite curative-intent treatment. The distribution of TRG, according to both the Mandard and Becker scoring systems, is summarized in Table 2. 

**Table 2:** The distribution of tumor regression grade, according to both the Mandard and Becker scoring systems

Mandard Score	n (%)	Becker Score	n (%)
Score 1	6 (15)	Score 1a	2 (5)
Score 2	4 (10)	Score 1b	5 (12.5)
Score 3	10 (25)	Score 2	15 (37.5)
Score 4	14 (35)	Score 3	18 (45)
Score 5	6 (15)	—	—

 The analysis of OS by the different groups of the Mandard and Becker scores is summarized in Table 3. 

**Table 3:** The overall survival of the different groups of the Mandard and Becker scores.

Scores	Mean Survival (months)	95%CI	Lower Limit	Upper Limit	p (Log Rank, global)	p (Log Rank, between score groups)
Mandard					p=0.496	1+2 vs. 3 vs. 4+5: 0.952
Score 1	49.2		34.5	63.8		
Score 2	28.7		7.7	49.8		
Score 3	23.7		18.9	28.3		
Score 4	43.6		30.7	56.5		
Score 5	39.4		22.9	55.8		
Becker					p=0.496	1/2 vs. 3: 0.752
Score 1	50.3		37.3	63.2		1 vs. 2/3: 0.374
Score 2	37.4		26.1	48.7		
Score 3	42.2		31.7	53.3		

CI: confidence interval.

### Performance of tumor regression grade scores

 There was no significant difference in OS based on Mandard’s TRG (p=0.496, p>0.05) or Becker’s TRG (p=0.496, p>0.05). However, the variation in OS rates across grades indicates these scores effectively stratify patients by survival. Table 3 summarizes this analysis. 

 By Mandard Score, OS was highest with TRG1 (49.2 months) and lowest with no response (TRG5: 39.4 months). Median OS was 48 months for TRG1, 18 months for TRG2, 24 months for TRG3, 21 months for TRG4, and 30.5 months for TRG5. By the Becker Score, the mean OS was highest for TRG1 (50.3 months) and lower for TRG2 (37.4 months). Median OS was 25.15 months (TRG1), 24 months (TRG2), and 54 months (TRG3). 

**Figure 1:**
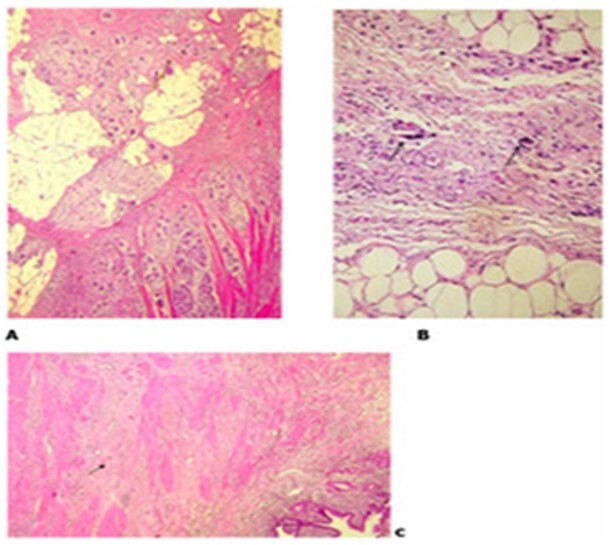
Histological images of tumor regression grades (TRGs) according to Mandard and Becker classifications. (A) TRG 3 (Mandard)/TRG 2 (Becker): carcinomatous proliferation associated with a mucinous component infiltrating the subserosa, with predominant fibrosis and mucinous areas over carcinomatous cells, which account for 30% of the tumor bed (hematoxylin and eosin ×100). (B) TGR 2 (Mandard)/TGR 1b (Becker): rare strands of residual carcinomatous cells (arrow) (hematoxylin & eosin ×200). (C) TRG 1 (Mandard)/TRG 1ª (Becker): absence of carcinomatous remnants. Gastric wall disrupted by fibrosis (arrow), with no residual carcinomatous cells (hematoxylin and eosin ×50).

 The study evaluated Mandard and Becker TRG scores for predicting 1-year OS. Mandard showed better homogeneity (likelihood ratio — LR: 1.116 vs. 0.418) and monotonicity (linear trend — LT: 0.6 vs. 0.3). Both scores had mediocre AUC values (Mandard: 0.568, Becker: 0.545) and failed to reliably predict 1-year OS (Figure 1). The Mandard TRG score had the highest positive LR+ for 5-year OS prediction (LR: 5.719 vs. 1.820). The Mandard score exhibited slightly higher linearity at 5 years compared to the Becker score (LT: 2.5 vs. 2.2). Both Mandard and Becker scores showed mediocre performance in predicting 5-year OS, with identical AUC values under the AUC curve of 0.606 (p=0.460, p>0.05). Neither score outperformed the other in discriminative ability. 

 Concluding, while the Mandard score had a higher LR+ and slightly better linearity, both scores demonstrated limited predictive performance for 5-year OS, with no significant difference in their discriminative ability. 

## DISCUSSION

 Histological response to neoadjuvant CT was assessed using the Mandard and Becker TRG systems. Both effectively stratified patients by survival: complete responders (TRG 1) had better outcomes, while non-responders had poorer prognoses. In our cohort, most patients had incomplete responses, with 35% classified as TRG 4 by Mandard and 45% as TRG 3 by Becker. When comparing the two systems, Mandard demonstrated slightly better homogeneity and linearity in predicting 1- and 5-year OS, although both showed similarly modest AUC values (0.606). 

 The MAGIC trial (2006) and ACCORD2 (2011) showed neoadjuvant CT improved staging, reduced tumor size, and increased 5-year survival (23–36.3% and 24–38%)^9,22^. 

 Neoadjuvant CT is now recommended for GC beyond stage IA, except in cases of stenosis or bleeding. Tunisia adopted it in 2014, with 2019 guidelines favoring the FLOT regimen. Benefits include reduced tumor size and higher resection rates^14,15^. 

 There are two types of response: parietal and cellular. Parietal response ("downstaging") is assessed by ypTNM (2017), categorizing good responders as ypT0-2N0 and poor respond ers as ypT3-4 and/or N1, though its predictive value is limited in multivariate analyses^4^. Cellular response evaluates residual tumor quantity, offering prognostic insights^18^. Mandard and Becker TRG systems are widely used, with some studies favoring TRG over ypT, though Schmidt et al. disagree^16^. 

 The Histological Regression Evaluation using Mandard TRG was introduced by Mandard et al. in 1994 for assessing tumor response in esophageal squamous cell carcinoma. This system grades responses from TRG 1 (complete response) to TRG 5 (no response), based on the fibrosis-to-viable tumor cell ratio. It is now widely applied to gastric and rectal can cers^12^. In our study, 35% of patients had TRG 4, and 25% had TRG 3. 

 The histological regression evaluation using Becker TRG was developed by Becker et al. in 2003. This three-score system evaluates tumor response based on the percentage of viable tumor cells: TRG 1 (minimal/no residual cells), TRG 2 (moderate residual cells), and TRG 3 (predominantly residual cells with minimal regression). It is also used for other gastrointestinal cancers^3^. A 2011 study on 480 gastric carcinomas identified TRG and postoperative lymph node status as independent prognostic factors^4^. In our study, TRG scores were TRG 3 in 45%, TRG 2 in 37.5%, and TRG 1 in 17.5% of patients. 

 TRG is considered by some authors as more prognostic than systems like pTNM. The AJCC acknowledges TRG for rectal carcinomas, though the UICC (International Union Agaisnt Cancer) does not^6^. In GADC, Mandard and Becker TRG systems are reproducible, with Becker favored by Svrcek et al.^18^ and Mandard et al.^12^ for survival prediction^5,18^. However, Smyth et al. found no independent impact of TRG on survival^17^. Complete regression indicates better survival, though partial regression’s significance is debated^20^. A 2022 study of 393 GADC patients showed higher survival for Mandard TRG 1-2 responders^7^, consistent with Portuguese findings using Becker’s system^11^. In our study, major responders (TRG 1) had a mean survival of 49.2 months (Mandard) and 50.3 months (Becker), compared to 39.4 months for non-responders (TRG 5). 

 An ideal TRG system should assess therapeutic response and prognosis reliably, but the optimal system remains debated. Tong et al. compared Mandard and Becker TRG scores for GADC patients, finding similar survival prediction performance with AUC values above 0.7^19^. Mandard slightly outperformed Becker for 1-year survival (AUC 0.68 vs. 0.65, p=0.513), while Becker had a marginal edge for 5-year survival (AUC 0.86 vs. 0.85, p=0.780), though differences were insignificant^19^. 

 Both systems were stronger for 3- and 5-year predictions than for 1-year predictions. In our study, Mandard showed higher LR+ and better linearity for 1- and 5-year survival, with AUCs of 0.568 and 0.606, respectively, while Becker’s were 0.545 and 0.606. Both systems exhibited moderate, comparable predictive capacities for long-term survival. 

 Both these scores show limitations. Inter and intra-observer variability significantly impacts TRG grading. Chetty et al. found simpler categories improved concordance among gastrointestinal pathologists5, and Svrcek’s study supported a three-category system for gastric carcinomas. The Mandard score groups "complete" responses without distinguishing minimal residual cells and imprecisely defines "rare" residual cells, while the Becker score oversimplifies partial regression and neglects lymph node changes, critical for therapeutic decisions^3,12^. Adopting a system like Sataloff and Chevallier’s, which integrates tumor and lymph node status, could enhance TRG assessment for OS and DFS^10^. 

 TRG scoring may improve prognostic accuracy when used in conjunction with ypTNM staging. 

## CONCLUSIONS

 This study confirms that the TRG classification is an independent prognostic factor in gastric cancer, particularly when combined with ypTNM staging. While Mandard shows slightly better performance, both Mandard and Becker scores demonstrate moderate and comparable predictive value, with no clear superiority. These findings highlight the need for a standardized system integrating nodal response and other key histological features to improve prognostic accuracy. 

## Data Availability

The Information regarding the investigation, methodology, and data analysis of the article is archived under the responsibility of the author.
